# Cord Blood-Based Neonatal Screening for Hemoglobinopathies in Northern Tunisia

**DOI:** 10.3390/ijns11040107

**Published:** 2025-11-14

**Authors:** Houyem Ouragini, Nizar Ben Halim, Sana Zitouni, Dorra Chaouachi, Imen Boudrigua, Naima Saidani, Imen Kraiem, Amira Ayachi, Salem Abbes, Mechaal Mourali, Samia Menif

**Affiliations:** 1 Laboratory of Molecular and Cellular Hematology, LR16IPT07, Institut Pasteur de Tunis, University of Tunis ElManar, Tunis 1002, Tunisia; 2 Laboratory of Physiopathology, Alimentation and Biomolecules, LR17ES03, Higher Institute of Biotechnology, Sidi Thabet, University of Manouba, Ariana 2020, Tunisia; benhalim_nizar@yahoo.fr; 3 Department of Gynecology and Obstetrics, Habib Bougatfa Hospital, Bizerte 7000, Tunisia

**Keywords:** newborn screening, thalassemia, sickle cell disease, capillary electrophoresis, molecular analysis

## Abstract

Hemoglobinopathies represent a major public health concern in Tunisia. Although early diagnosis is essential, systemic neonatal screening has not yet been implemented at the national level. We conducted a screening study in Northern Tunisia (Bizerte region) using cord blood samples. Complete blood counts and hemoglobin analysis by capillary electrophoresis were performed. Samples showing abnormal profiles (HbBart’s, HbS, HbC, or HbA < 20%) underwent molecular testing. Correlations between hematological parameters, hemoglobin fractions, and β mutation types were assessed. Among 328 neonatal cord blood samples analyzed, we detected 3 silent α^+^-thalassemia, 6 β^+^-thalassemia traits, 3 β^0^-thalassemia traits, 7 HbS traits, 2 HbC traits, and 1 compound heterozygous for α^+^-thalassemia/HbC. No homozygous cases were identified. The heterozygous frequency was estimated at 1.2%, 2.7%, and 2.1% for α-thalassemia, β-thalassemia, and sickle cell disease, respectively. HbF levels were significantly associated with the β-thalassemia trait. This study represents the first hemoglobinopathy screening in Northern Tunisia using cord blood, highlighting the feasibility and reliability of this approach. While pilot programs have already been initiated in some regions, our findings reinforce the need for broader implementation to ensure early and accurate diagnosis across the country.

## 1. Introduction

Hemoglobinopathies are among the most common monogenic diseases and one of the world’s major health problems [1]. Migration from regions of high prevalence, especially in the Mediterranean and sub-Saharan Africa regions, has resulted in a rise in patient numbers all over the world [2]. This group of inherited disorders is caused by structural abnormalities or impaired production of hemoglobin (Hb), affecting its function. The clinical presentation ranges from asymptomatic carrier states to severe, life-threatening anemia, and multi-organ complications [3].

In Tunisia, the three major common sub-types of hemoglobinopathies are α-thalassemia, β-thalassemia, and sickle cell disease (SCD) [4].

α-thalassemia is usually due to deletions, and less commonly point mutations with a marked genetic heterogeneity. This reduces (α^+^) or abolishes (α^0^) the expression of α-globin genes *HBA1*/*HBA2* [5]. Based on the number of affected genes, the following four clinical forms are recognized: silent carrier (with one gene affected), α-thalassemia trait (with two affected genes), HbH disease (with three affected genes), and HbBart’s hydrops fetalis (all the α-globin genes are affected). The severity ranges from asymptomatic (silent carrier) to fatal in utero, with intermediate forms showing varying degrees of microcytic, hypochromic anemia [6,7].

Unlike α-thalassemia, β-thalassemia is mainly caused by mutation in the *HBB* gene, resulting in reduced (β^+^) or absent (β^0^) synthesis of β-globin chains of hemoglobin [8]. Over 300 mutations have been reported worldwide [9]. β-thalassemia manifests in a spectrum of clinical phenotypes, from the asymptomatic β-thalassemia trait to the severe, transfusion-dependent form known as β-thalassemia major. Individuals with this form typically develop severe anemia within the first two years of life, requiring regular blood transfusions and iron chelation therapy. A milder intermediate form, β-thalassemia intermedia, also exists [10].

SCD is caused by the Glu6Val mutation in the *HBB* gene, leading to the production of abnormal hemoglobin S (HbS) [11]. Under deoxygenated conditions, HbS polymerizes, giving the red blood cells a characteristic sickle shape. It results in chronic hemolytic anemia, severe pain crises, and increased risk of complications like vaso-occlusive crises, and progressive organ damage [12]. Although SCD is caused by a unique and single mutation, its clinical severity varies widely, influenced by factors such as fetal hemoglobin levels, co-inheritance of α-thalassemia, and genetic modifiers, as seen in β-thalassemia [13].

Although hematopoietic stem cell transplantation and emerging gene therapies offer curative potential for hemoglobinopathies, their use remains limited by availability, high cost, and associated risks, particularly in many low- and middle-income countries such as Tunisia. Consequently, treatments such as hydroxyurea, blood transfusions, and supportive care remain the mainstay of management, effectively controlling symptoms and improving patients’ quality of life [14]. In this context, neonatal screening for hemoglobinopathies is essential. Indeed, early diagnosis of these conditions allows for early initiation of appropriate interventions, including prophylactic antibiotics, vaccination, transfusion programs, and parental education. These measures substantially reduce disease-related complications and improve clinical outcomes [15,16].

Neonatal screening for hemoglobinopathies, typically performed using cord blood (CB) or heel-prick samples, is well-established in many countries [17,18,19,20,21,22,23]. In Tunisia, however, no such program exists, despite the high prevalence of hemoglobinopathies, which ranges from 1.2% to 12.5% reflecting marked regional heterogeneity [4]. Here, we present the results of the first study on CB screening in northern Tunisia, providing evidence to support for the implementation of a national program to address these disabling disorders.

## 2. Materials and Methods

### 2.1. Samples Collection

This study was approved by the Pasteur Institute’s Biomedical Ethics Committee, Tunisia (no.:2018/.5/I/LR16IPT07). Before starting the neonatal screening, parents were provided with an information leaflet by healthcare professionals involved in prenatal or perinatal care (physicians or midwives), detailing hemoglobinopathies, the benefits of screening, and their right to freely consent without consequences for routine healthcare procedures. Informed written consent was obtained from each pregnant woman prior to delivery.

Neonatal screening was conducted at Bizerte’s “Habib Bougatfa” hospital, North Tunisia, over two periods, April–May 2019 and January–May 2020, for a total of 22 weeks. The CB was carefully collected by midwives from the umbilical vein immediately clamped after the baby delivery to avoid contamination by maternal blood, into ethylenediamine tetra-acetic acid (EDTA) tubes. Samples were stored less than 24 h before laboratory analyses.

The inclusion criteria were gestational age greater than 36 weeks and vaginal delivery, consent from one of the parents; the exclusion criteria were complications during pregnancy or delivery for mother or neonate and intrauterine transfusions. The non-inclusion criteria were samples less than 2 mL, storage more than 24 h.

Gender of the newborn, weeks of gestation, birth weight, region parent’s origin, familial history of hematological diseases, and consanguinity were recorded for epidemiological analysis.

### 2.2. Laboratory Analyses

#### 2.2.1. Hematological Analyses

Complete Blood Count (CBC) was automatically obtained using an automated blood cell counter (Coulter Counter ABX Micro-60-OTR; ABX Diagnostics, Montpellier, France). The parameters included Hb level, red blood cell (RBC), hematocrit (Hct), mean corpuscular volume (MCV), mean corpuscular Hb (MCH), mean corpuscular Hb concentration, red cell distribution width (RDW), red cell, white cell, and platelet counts.

#### 2.2.2. Hemoglobin Electrophoresis

The Hb profiles were determined by capillary electrophoresis (CE) (Capillarys 2, SEBIA, Lisses, France) using blood control HbAF (Sebia, PN4777).

#### 2.2.3. Molecular Analyses

Genomic DNA was extracted from leukocytes of whole blood following the standard phenol–chloroform method [24]. For the *HBB* gene (NG_000007.3), the amplification was performed as previously described [25]. PCR/RFLP with restriction enzyme DdeI or BseRI were used for the sample with HbS and HbC, respectively. DNA sequencing was performed using the ABI Prism 310 Genetic Analyzer (Applied Biosystems, Ontario, CA, USA). Mutation has been named according to HGVS recommendations and reviewed in the hbvar database. Gene Bank reference sequences used in this study are NM_000518.4 and NP_000509.1.

Samples with HbBart’s peak underwent GAP PCR in order to identify the most frequent deletion as previously described [26].

### 2.3. Statistical Analyses

All statistical analyses were performed using R software (version 4.5.1) with RStudio (version 2025.5.1.513) as the integrated development environment. *p* < 0.05 was considered statistically significant.

Descriptive statistics were summarized as mean ± standard deviation (SD) for all parameters to maintain consistency in data presentation. The normality of the quantitative variables was tested with the Shapiro–Wilk test. When all groups showed normally distributed data (*p* > 0.05), comparisons between groups were conducted using one-way ANOVA followed by Tukey’s Honest Significant Difference (Tukey HSD) test for pairwise comparisons. If any group deviated from normality, the non-parametric Kruskal–Wallis test was applied, followed by Dunn’s test with Benjamini–Hochberg correction for multiple comparisons. Boxplots were generated to visualize the distribution of data and the results of pairwise statistical comparisons.

## 3. Results

### 3.1. Study Population and Initial Screening

**Figure 1:**
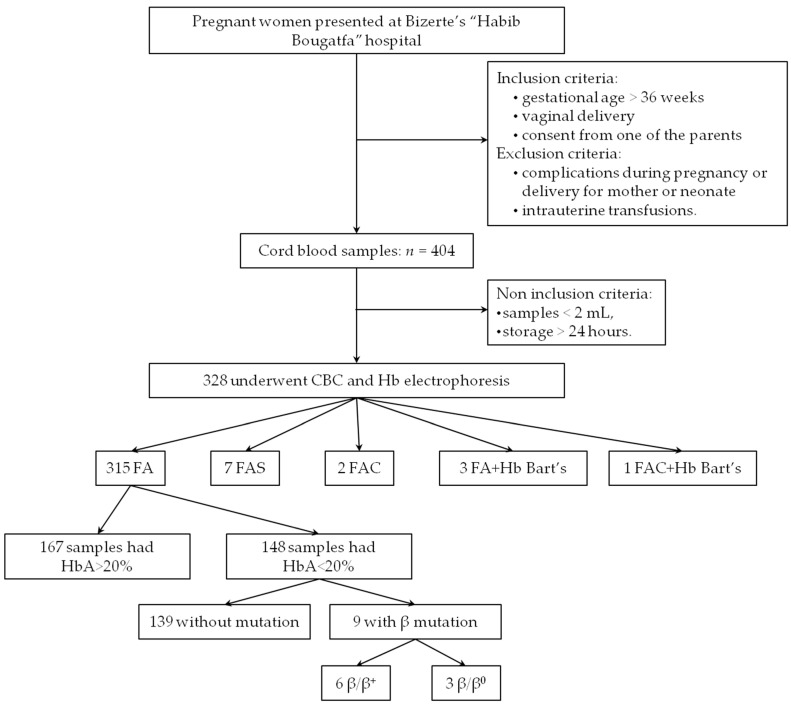
Flow diagram of the present study. CBC: complete blood count; Hb: hemoglobin. All the samples with HbBart’s carried the −α3.7/αα genotype.

During the 22-week-period screening, 404 cord bloods were collected. A total of 76 were eliminated because they met at least one exclusion criterion (Figure 1). The 328 remaining samples underwent hematological analysis (CBC and Hb electrophoresis).

The mothers’ ages were 29.62 ± 5.41 years. All samples were from vaginal deliveries and the birth weights of the newborns were 3.34 ± 0.42 kg. The newborns were all full-term, with an average gestational age of 39.47 ± 1.24 weeks (77.14% were born between 39 and 40 weeks). The sex ratio was M/F = 1.06.

Based on the Hb electrophoresis analysis, the majority (96%) of the samples were FA phenotype; among them 53% (*n* = 167) had HbA > 20%. No HbSS, nor β-thalassemia major (with a total absence of HbA) were identified (Figure 1, Table 1).

A total of 161 CB samples underwent molecular analysis; all were suspected to be heterozygous for a mutation, based either on HbA levels <20% or on the presence of an additional peak on CE (HbBart’s, HbS, or HbC).

### 3.2. Abnormal Patterns

Hemoglobin electrophoresis revealed an extra peak for 13 newborns: 7 had HbS (7/328: 2%), 3 had HbBart’s characteristic peak (3/328: 0.9%), 2 had HbC (2/328: 0.6%), and one was suspected to be heterozygous composite HbBart’s associated with HbC (Figure 1, Table 1). For heterozygous HbS and HbC, the phenotype was confirmed by PCR/RFLP; and for silent α-thalassemia, GAP PCR revealed a 3.7 deletion in all the 4 cases (the 3 heterozygous newborns with silent α-thalassemia, and the heterozygous composite newborn HbBart’s/HbC). Their hematological data are summarized in Table 2.

### 3.3. Detection of β-Thalassemia Carriers

Unlike in adults, where the β-thalassemia trait can be identified using well-established hematological and electrophoretic parameters, no standardized criteria exist for CB. Therefore, the identification of carriers in this study was based on the literature. Given the limited number of studies on this topic and the absence of HbA threshold for CB, we opted to apply a broad cutoff of 20% [27,28]. As a result, 147 newborns with an HbA < 20% underwent direct sequencing of the entire *HBB* gene to identify potential mutations.

Results revealed 6 heterozygous mutations in 9 newborns: 6 β^+^ mutations and 3 β^0^ mutations (Figure 1, Table 3). Thus, the carrier frequency of β-thalassemia was 2.74% (9/328). None of them presented HbA2.

IVS-I-110 G>A (*HBB*: c.93-21G>A) was the most frequently encountered mutation with a constituent ratio of 33% (3/9), followed by Codon 39 C>T (*HBB*: c.118C>T) (22%; 2/9) (Table 3).

### 3.4. Hematological Comparisons Between Groups

**Figure 2:**
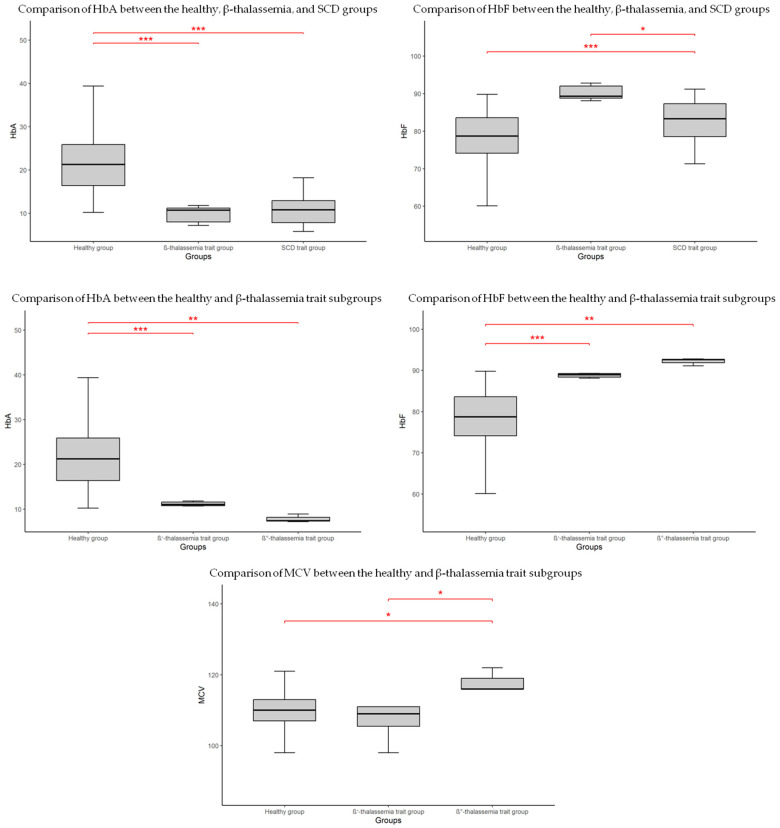
Boxplot showing significant differences in hematological parameters between groups. * *p* < 0.05; ** *p* < 0.01; *** *p* < 0.001.

Comparison of CBC parameters between the healthy group, the β-thalassemia trait group, and the SCD trait group revealed no significant differences (*p* > 0.05). In terms of HbF levels, significant differences were observed between the healthy group and both the β-thalassemia trait and SCD trait groups (*p* < 0.001). No significant difference was found between the β-thalassemia and SCD newborns. HbA levels were lower in the β-thalassemia trait group compared to both the healthy and SCD trait groups (*p* < 0.001 for both comparisons). (Figure 2, Table 2).

Despite the limited number of identified carriers, the β-thalassemia cohort was further subdivided into two subgroups based on mutation type, to explore potential associations (Table 2). In this sub-group, as shown in the boxplot (Figure 2), HbF levels were significantly higher in both β^+^-thalassemia (*p* < 0.001) and β^0^-thalassemia (*p* < 0.01) compared to healthy newborns. Similarly, HbA levels were reduced in both β-thalassemia subgroups relative to the healthy group. Among all CBC parameters, only MCV showed a statistically significant difference, being higher in the β^0^-thalassemia group compared to both the healthy group and the β^+^-thalassemia group (*p* < 0.05 for both comparisons).

## 4. Discussion

Hemoglobinopathies, a group of inherited blood disorders affecting hemoglobin structure or production, pose significant public health challenges worldwide. In Tunisia, where consanguineous marriages are common and estimates range from 40% to 49% [29], hemoglobinopathies remain particularly common in certain regions, necessitating effective diagnostic and management strategies [30]. Although the exact incidence of hemoglobinopathies is unknown, it has been estimated that 4.48% of the Tunisian population are carriers, with 5.48% heterozygous for α-thalasemia mutations, and 2.2% heterozygous for β-thalassemia mutations [31,32]. SCD prevalence among the population reached a rate of 1.89% [4]. Out of 328 neonatal CB analyzed in our study, there were 7 phenotypes: 308 healthy, 3 heterozygous α^+^-thalassemia, 6 heterozygous β^+^-thalassemia, 3 heterozygous β^0^-thalassemia, 7 heterozygous HbS, 2 heterozygous HbC, and 1 compound heterozygous for α^+^-thalassemia/HbC. No homozygous or severe cases were identified; therefore, no specific communication or counseling was provided to the parents. The heterozygous frequency was estimated at 1.2%, 2.7%, and 2.1% for α-thalassemia, β-thalassemia, and SCD, respectively. The difference with the reported prevalence may be partly explained by the limited sample size. Regional genetic variation could also contribute to the observed discrepancies, particularly as the region assessed in our study was investigated for the first time.

Early detection through neonatal screening offers a critical step in reducing the burden of hemoglobinopathies, yet such programs remain insufficiently developed in our country. In SCD, timely diagnosis combined with comprehensive care, including prophylactic antibiotics, vaccination, hydroxyurea therapy, and transfusion support, significantly improve prognosis [15]. Similarly, in β-thalassemia major, where first symptoms usually appear between 6 and 24 months, early recognition and the initiation of a regular transfusion program can normalize growth and development at least through the first decade of life [33]. These examples highlight the potential of CB screening as an effective strategy to identify both affected individuals and carriers at birth.

CB sampling offers several key advantages for neonatal screening: (1) it is less invasive and generally more accepted by families than heel pricks or peripheral blood sampling; (2) it provides a large volume, which allows multiple analyses such as DNA testing; (3) it ensures high quality and stability compared to dry samples; (4) it allows results shortly after birth [34,35]. The major disadvantage of the use of this type of sample is probably the risk of contamination by maternal blood. Wolff et al. demonstrated that maternal contamination would need to be very high to alter the fetal hemoglobin profile and suggested that an HbA level above 40% is a telltale sign [34]. In our study, all the samples had HbA < 40% (range between 2.1 and 38.9%), suggesting that CB was not contaminated with maternal blood and was suitable for testing. Once sample integrity was ensured, analysis was carried out with the Capillarys Hemoglobin Kit (Sebia), a fully automated system that allows easy interpretation of the Hb fractions, as well as the detection of all major and most minor haemoglobinopathies [36]. As recommended, a second-line test that used a different methodology, here molecular analysis, was necessary to confirm the results obtained by CE [34,37].

**Figure 3:**
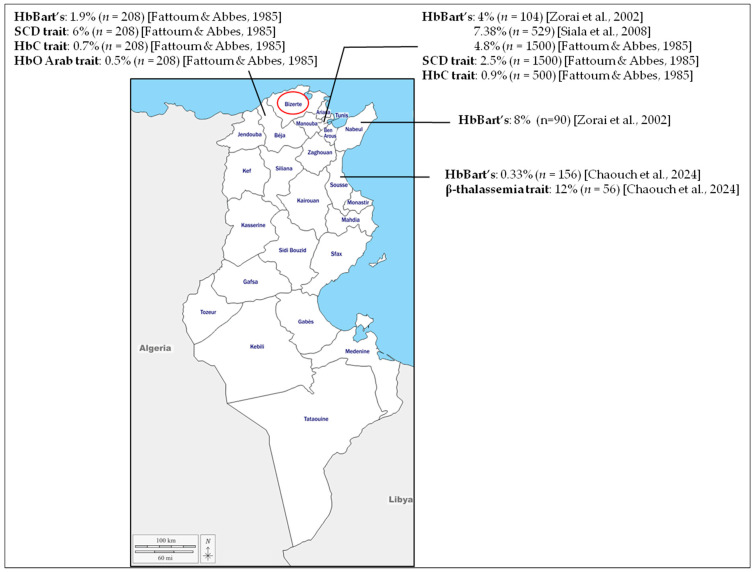
Results of the previous cord-blood neonatal screening performed in Tunisia. Neonatal screening using cord blood has been reported in only four regions: Beja, Tunis, Nabeul, and Sousse. The Bizerte region, investigated in the present study, is outlined in red. It should be noted that patients sampled in Tunis may originate from different regions across the country [31,40,41,42].

Screening of CB for hemoglobinopathies was reported first in 1972 [38,39]. It was focused primarily on SCD due to its severity and high frequency, particularly among populations of sub-Saharan African origin. The objective was to achieve early diagnosis for homozygous hemoglobin S disease, thereby enabling timely interventions to prevent severe complications and reduce disease-related mortality. Since then, the use of CB screening has expanded to encompass other hemoglobinopathies [16,22]. In Tunisia, only a few neonatal screening initiatives based on CB have been conducted, and these remain limited to specific regions. Most of these studies have focused on α-thalassemia, with reported HbBart’s prevalence ranging from 0.33% to 8% [4,31,40,41,42]. To our knowledge, only one study on β-thalassemia using CB has been conducted in Tunisia, reporting a carrier frequency of 12% in the central region [42]. Variants S, C, and O Arab were also identified in these screenings [40] (Figure 3).

Deletions affecting α-globin genes are the primary cause of α-thalassemia, as they reduce or completely suppress α-globin production. Among the resulting phenotypes, the silent carrier form, caused by the loss of a single gene (-α/αα), is typically asymptomatic and produces little hematological alterations, making it difficult to detect [6]. Alone, it rarely requires treatment, but when associated with other conditions, it can complicate diagnosis and influence clinical outcomes, hence the importance for its accurate diagnosis [7,43]. Several studies have demonstrated a correlation between the HbBart’s level and the number of deleted α-globin genes [44,45]. While a threshold of 2% has been established in CB for identifying cases with two gene deletions, defining a cut-off for detecting single-gene deletions remains challenging [34]. This difficulty is further complicated by reports of elevated HbBart’s levels in individuals without α-globin gene deletions, as well as cases lacking detectable HbBart’s despite the presence of α-globin defect [46]. Such discordances are likely influenced by the hemoglobin quantification methods and the molecular analysis. Nevertheless, some studies have proposed a threshold of 0.2% or 0.3% for the detection of heterozygous α-thalassemia in CB [47,48]. In our study, 4 cases showed HbBart’s peak at a range of 0.3 to 1.9%; one of them is associated with the benign variant HbC, an association that had already been reported in a previous Tunisian neonatal screening study [41]. Molecular analysis revealed that all the newborns have the 3.7 deletion, which is the most prevalent α-globin gene defect in Tunisia [31,41]. Due to the limited number of identified cases, it was not possible to establish reliable HbBart’s cut-off points.

Establishing an appropriate HbA cut-off is also essential for effective β-thalassemia screening using CB. However, HbA levels are known to increase progressively with gestational age, reaching approximately 70–80% by the age of 6 months. This physiological progression explains the limited number of cord blood-based neonatal screening for β-thalassemia. In our cohort, 77.14% were delivered between 39 and 40 weeks of gestation. Previous studies using CE method have reported HbA levels around 20.5% in healthy full-term neonates, with median values ranging from 19 to 21.9% in CB collected after 39 and 40 weeks [27,34]. These findings suggest that HbA values below this range may indicate the presence of a β-thalassemia mutation. Although it has been suggested that HbA levels in CB are not a reliable parameter for screening heterozygous β-thalassemia [46], the few published studies in this area use markedly different thresholds: as low as 6% [34], 10% [28], or 15% [27]. As this is the first study conducted in the northern region of Tunisia, we opted for a relatively high cut-off of 20% in order to minimize the risk of missing carriers of β-thalassemia mutations. Among the 47% FA samples with HbA < 20%, 9 presented a heterozygous mutation in the *HBB* gene with an HbA level ranging from 7.2% to 11.8%. A total of six mutations have been identified with Cd39 and IVS-I-100, the most frequent as reported in the Tunisian population [4]. To date, we have no evidence of false-negative results. The level of HbA may represent a promising parameter for identifying β-thalassemia carriers. Using a low threshold increases the likelihood of detecting all carriers and even mild intermedia cases. However, this approach may fail to identify heterozygous with higher HbA values [27]. Although our preliminary results are encouraging, they are based on a limited cohort and should be validated in a larger population to assess the reliability and effectiveness of this strategy using different HbA cut-offs.

During our neonatal screening, seven newborns were identified as heterozygous carriers HbAS. No cases of homozygous HbS variant were detected. The identification of heterozygous carriers at birth remains of epidemiological relevance, as it provides insights into the distribution of the sickle cell gene in the population, even though these individuals are clinically asymptomatic.

Only HbC was identified among the rare Hb variants known in the Tunisian population [49], and it was found in two cases. This may reflect the relatively higher prevalence of the HbC variant in the studied region compared to other rare variants or could simply be related to the limited size analysis.

Although no severe cases were identified in this study, the results highlight the importance of establishing a structured neonatal screening program for hemoglobinopathies in Tunisia. To date, and to the best of our knowledge, no nationwide neonatal screening for hereditary diseases has been implemented. Hemoglobinopathy screening is restricted to at-risk families, in whom carrier detection and prenatal diagnosis, including molecular testing, are performed [50]. Establishing a national neonatal screening program would represent a major advancement, enabling early diagnosis, timely clinical management, and appropriate family counseling, thereby reducing disease-related morbidity and healthcare burden. However, its implementation faces several challenges, including the need for adequate infrastructure, trained personnel, sustainable funding, and increased public and professional awareness. To move toward this goal, priority should be given to developing national guidelines, ensuring long-term sustainability, and strengthening laboratory and data infrastructures to support large-scale implementation. In the long term, such a program could be integrated into a broader framework encompassing hemoglobinopathies and other severe congenital disorders, thus optimizing resources and maximizing the overall impact on public health.

## 5. Conclusions

Our findings underscore the importance of integrating neonatal CB screening into a national health strategy for hemoglobinopathies in Tunisia, where prevalence is high and consanguinity remains common. While small pilot programs have been shown feasibility, broader implementation is limited by financial, logistical, and infrastructural challenges. Molecular confirmation of mutations adds value by enabling accurate carrier detection and targeted prevention. Strong governmental support and coordinated efforts with healthcare providers and patient associations are essential to ensure sustainability, effective follow-up, and improve outcomes for affected families, paving the way for the establishment of a national neonatal screening program in Tunisia.

## Figures and Tables

**Table 1:** Hemoglobin pattern in our cohort based on CE.

Hemoglobin Pattern	Frequency (*n*)	Percentage (%)
FA *	315	96
FAS	7	2
FAC	2	0.6
FA Bart’s	3	0.9
FAC Bart’s	1	0.3
Total	328	100

*: samples with FA phenotype had HbA > 20% (167) and HbA < 20% (148).

**Table 2:** Hematological and hemoglobin analysis data in correlation with genotype. All parameters were demonstrated as mean ± SD.

Genotype	Expected Phenotype (*n*)	Hb (g/dL)	RBC (10^6^/µL)	Hct (%)	MCV (fL)	MCH (ρg)	RDW (%)	HbF (%)	HbA (%)	HbA2 (%)	HbBart’s (%)	HbS (%)	HbC (%)
αα/αα β/β	Healthy (308)	14.84 ± 2.34	4.20 ± 0.92	45.66 ± 7.48	109.64 ± 5.08	32.60 ± 0.84	11.62 ± 0.83	78.14 ± 7.25	21.81 ± 7.21	0.06 ± 0.15	-	-	-
−α^3.7^/αα	Silent α-thalassemia (3) *	14.7 13 16.2	4.74 4.62 4.93	46.6 41.4 51.3	102 90 104	31.3 13.4 13.6	11.7 12.9 11.3	81.2 64.8 76.9	18.3 33.3 22.8	0	0.5 1.9 0.3	-	-
β/β^+/0^	β-thalassemia trait (9)	15.99 ± 2.60	4.41 ± 0.73	49.29 ± 8.46	111.89 ± 7.79	32.49 ± 0.56	12.09 ± 1.41	90.22 ± 1.90	9.76 ± 1.88	0	-	-	-
β/β^+^	β^+^-thalassemia trait (6)	15.48 ± 3.01	4.40 ± 0.89	47.80 ± 9.81	108.83 ± 7.65	32.48 ± 0.68	12.55 ± 1.48	89.25 ± 1.43	10.72 ± 1.40	0	-	-	-
β/β^0^	β^0^-thalassemia trait (3) *	18.3 15.4 17.3	4.91 4.05 4.36	56.8 46.8 53.2	116 116 122	32.2 32.8 32.5	11.5 11.7 10.3	92.8 92.6 91.1	7.2 7.4 8.9	0	-	-	-
β/β^S^	HbS trait (7)	15.61 ± 2.96	4.45 ± 0.87	48.23 ± 9.92	108.29 ± 3.90	32.47 ± 0.78	11.74 ± 0.80	82.50 ± 7.16	10.90 ± 4.35	0	-	6.60 ± 2.86	-
β/β^C^	HbC trait (2) *	15.9 15.7	4.67 4.59	50.2 49.8	107 105	31.7 31.8	12.3 12.1	64.4 82.8	21 10.5	0	-	-	13.6 6.7
β/β^C^, −α^3.7^/αα	HbC trait, silent α-thalassemia (1) *	10.2	3.7	33.2	90	30.7	12.9	81.4	2.1	0	0.8	-	15.7

Hb: hemoglobin, RBC: red blood cell, Hct: hematocrit, MCV: mean corpuscular volume, MCH: mean corpuscular hemoglobin, RDW: red cell distribution width. *: less than 3 samples in this category. Absolute results are depicted instead of the median and percentiles.

**Table 3:** β-thalassemia mutations identified during our newborn screening.

Mutation	HGVS Nomenclature	Mutation Type	Number of Carriers (n)	Ratio (%)	Hb (g/dL)	MCV (fL)	MCH (ρg)	HbF (%)	HbA (%)
IVS-I-110 G>A	c.93-21G>A	β+	3	33.3	16.2 12.7 12.8	11 121 98	32.1 33 33.1	92 89.1 88.1	8 10.9 11.7
Cd39 C>T	c.118C>T	β0	2	22.2	15.4 17.3	116 122	37.9 39.7	92.6 91.1	7.4 8.9
PolyA (T>A)	c.*110T>A	β+	1	1.1	15.5	105	31.5	88.8	11.2
IVS-I-5 (G>A)	c.92+5G>A	β+	1	1.1	14.8	107	33.1	89.3	10.7
IVS-I-6 T>C	c.92+6T>C	β+	1	1.1	20.9	111	32.1	88.2	11.8
Cd 6 (−A)	c.20delA	β0	1	1.1	18.3	116	37.3	92.2	7.2

Hb: hemoglobin, MCV: mean corpuscular volume, MCH: mean corpuscular hemoglobin. (*) in c.*110T>A follows the HGVS nomenclature, where it indicates that the variant is located in the 3’UTR, downstream of the translation termination codon.

## Data Availability

The data presented in this study are available on request from the corresponding author.

## Abbreviations

The following abbreviations are used in this manuscript:

Hb	Hemoglobin
SCD	Sickle Cell Disease
CB	Cord Blood
CBC	Complete Blood Count
RBC	Red Blood Cell
Hct	Hematocrit
MCV	Mean Corpuscular Volume
MCH	Mean Corpuscular Hemoglobin
RDW	Red Cell Distribution Width
CE	Capillary Electrophoresis
SD	Standard Deviation
